# CD3+ and CD8+ T cell-based immune cell score as a prognostic factor in clear-cell renal cell carcinoma

**DOI:** 10.2340/1651-226X.2024.19690

**Published:** 2024-03-28

**Authors:** Jonne Åkerla, Olli Helminen, Juha P. Väyrynen, Anne Parkkinen, Hilma Järvenpää, Jan Böhm, Maarit Ahtiainen, Heikki Seikkula

**Affiliations:** a Department of Urology, Tampere University Hospital, Tampere, Finland; b Cancer and Translational Medicine Research Unit, Medical Research Centre, University of Oulu and Oulu University Hospital, Oulu, Finland; c Department of Gastrointestinal Surgery, Oulu University Hospital, Oulu, Finland; d Department of Surgery, Hospital Nova of Central Finland, Jyväskylä, Finland; e Department of Pathology, Hospital Nova of Central Finland, Jyväskylä, Finland

**Keywords:** Carcinoma, renal cell, immunohistochemistry, immune cell score, CD3, CD8, Prognosis

## Abstract

**Background and purpose:**

Immunoscore^®^ is a prognostic parameter based on densities of lymphocyte populations in the tumor center and invasive margin. Immunoscore^®^ is validated in colorectal cancer as a high Immunoscore^®^ is associated with longer survival. Previous studies have suggested that Immunoscore^®^ may also predict oncological outcomes in clear-cell renal cell carcinoma (ccRCC). This study aims to assess the prognostic role of immune cell score in ccRCC.

**Material and methods:**

All patients with ccRCC undergoing surgery between 2007 and 2020 in Central Finland Central Hospital were retrospectively identified. CD3+ and CD8+ cell densities were calculated from tissue samples to determine the immune cell score using Immunoscore^®^ principles. Receiver-operating characteristic analysis, Kaplan–Meier survival curve, and Cox regression were used to evaluate the association between immune cell score and survival.

**Results:**

A total of 203 patients (mean age 66.5 years) were identified. The median follow-up time was 6.2 years. Based on the immune cell score, the patients were divided into three groups: low, intermediate, and high. In Cox regression analysis, adjusted with age, sex, and Charlson Comorbidity Index, no significant differences in disease-specific mortality were observed among the three groups. The hazard ratios (HRs) for disease-specific mortality were 0.93 (95% confidence interval [CI] 0.48–1.79) and 1.12 (0.52–2.37) for intermediate- and high-immune cell score groups when compared to low-immune cell score group, respectively.

**Interpretation:**

This study found no association between immune cell score and survival. These results indicate that immune cell score may not serve as a prognostic tool in ccRCC.

## Introduction

Renal cell carcinoma (RCC) comprises approximately 3% of all cancers and 1.8% of all cancer deaths worldwide [1]. RCC is more common among men as the relative risk for RCC is 1.7 for men compared to women [2]. There is a broad spectrum of histopathological entities of RCC, but clear-cell RCC (ccRCC) is the most common histological subtype and accounts for 70% of all RCC [3].

At present, RCC is mainly prognostically classified by the Tumor Node Metastasis (TNM) staging system [4, 5]. The 5-year survival rate for localized RCC is high (92.6%) [6]. In contrast, RCC with distant metastasis has a poor 5-year survival rate of 14%. However, clinical outcome can vary substantially among tumors of the same stage. Therefore, prognostic models integrating multiple prognostic factors have been developed for both local and metastatic diseases [7]. Nevertheless, none of those used in clinical practice includes information on biological differences in the host immune response.

The tumor microenvironment encompasses a diverse array of factors, including signaling molecules, endothelial cells, fibroblasts, and lymphoid and myeloid cells recruited from nearby tissues or derived from precursors that originate in the bone marrow [8]. Immunoscore^®^ is a tumor prognostic parameter that is based on densities of lymphocyte populations, in particular CD3+ and CD8+ T-cells in the tumor center and invasive margin [9, 10]. Immunoscore^®^ is internationally validated in colorectal cancer and is proposed to be part of TNM-Immune classification [11]. In colorectal cancer, high Immunoscore^®^ associates with longer survival. Furthermore, there is evidence that scoring systems similar to Immunoscore^®^ may harbor prognostic value in other cancer types [12, 13]. In ccRCC, the prognostic role of Immunoscore^®^ is unclear even if there is some evidence that T cell densities may be useful in predicting oncological outcomes after surgery [14].

The aim of the present study is to evaluate the prognostic role of immune cell score (based on CD3+ and CD8+ cell densities according to the principles of the Immunoscore^®^ assay for colorectal cancer) in a cohort of surgically treated ccRCC patients with local or metastatic disease.

## Material and methods

### Patients

All patients with histologically confirmed ccRCC undergoing surgery (nephrectomy or partial nephrectomy) in Central Finland Central Hospital from January 01, 2007 to July 30, 2020 were identified from a database of surgical and pathological records. Clinical data were obtained through patient records. This study benefited from samples/data from Central Finland Biobank, Jyväskylä, Finland. Tumors were classified according to the 8th edition of the UICC/AJCC TNM categories [4]. Survival data were obtained through Statistics Finland, which maintains the Cause of Death Registry. The hospital district approved the study. The use of patient data and study samples was approved by the Ethics Committee and by the Finnish Medicines Agency (FIMEA).

### Immunohistochemical analyses

Prospectively collected diagnostic hematoxylin and eosin stained histological samples of the tumors were retrieved and digitalized using a NanoZoomer-XR (Hamamatsu Photonics, Hamamatsu City, Japan) slide scanner with a 20× objective. Tissue cores of 1 mm diameter were punched into tissue microarrays using TMA Master II (3DHistech Ltd., Budapest, Hungary). For each tumor, two cores from the tumor center and two from the invasive margin were selected, which was guided by digital images of hematoxylin–eosin-stained tumor sections to represent the average histological findings of the tumors. The tissue microarray blocks were cut into 3.5 µm-thick sections for immunohistochemistry.

CD3 and CD8 immunohistochemistry were conducted with Leica Bond III automated IHC stainer (Leica Biosystems, Buffalo Grove, IL, USA). The slides were baked, deparaffinized, and rehydrated. The antigen retrieval was conducted with Bond Epitope Retrieval Solution 2 (Leica Biosystems, AR9640, 100°C, 20 min). The primary antibodies (CD3, LN10, Leica Biosystems, PA0553, ready-to-use; CD8, 4B11, Leica Biosystems, PA0183, ready-to-use) were incubated for 30 min and subsequently visualized using the Bond Polymer Refine Detection kit (Leica Biosystems, DS9800).

The slides were scanned with NanoZoomer-XR and analyzed with QuPath [15]. The tissue microarray cores were separated using the *TMA dearrayer* function. Only cores that contained tumor cells and were free of artifacts were included. The densities of CD3+ and CD8+ cells were counted using the *Fast cell counts* function. A script was written to enable batch analysis of all images. To calculate immune cell score according to the principles of Immunoscore^®^, the CD3+ and CD8+ lymphocyte densities in the invasive margin and tumor center were converted into percentiles across all samples, resulting in four percentile scores for each tumor. The mean of the four percentile scores was calculated, and it was categorized as low (a mean percentile of ≤ 25%), intermediate (between > 25% and ≤ 70%), and high (> 70%). Core-to-core correlations (Spearman correlation coefficients) for tumors with more than one core analyzed (CD3 tumor center, *N* = 196; CD3 invasive margin, *N* = 169; CD8 tumor center, *N* = 201; CD8 invasive margin, *N* = 172) varied between 0.60 (CD8 invasive margin) and 0.71 (CD8 tumor center), supporting the adequacy of the tissue microarray method in evaluating the immune cell infiltrates.

### Statistical analyses

Chi-square test was used for group comparison in categorical variables. Kruskal–Wallis test was used for continuous variable group comparison. Groups were formed based on previously suggested cutoff values [11] resulting with low, intermediate, and high groups. Receiver operating characteristic (ROC) analysis was used for additional cutoff determination. A Kaplan–Meier survival curve was made with log rank test for statistical comparison. The estimates for hazard ratios (HRs) with 95% confidence intervals (CIs) were calculated using Cox regression. For multivariate analysis of immune cell score, the Cox regression model was adjusted for sex (male or female), age (as a continuous variable), and Charlson Comorbidity Index (CCI) (0, 1, 2, or ≥ 3). Statistical analysis was performed using IBM SPSS Version 29.

## Results

A total of 203 patients were identified and included in this study. Demographic information of the study population is summarized in Table 1. There were no significant differences between the three immune cell score groups. The mean age was 66.5 years, and 54.2% were men. CCI was 0 (*n* = 87) or 1 (*n* = 58) in the majority of the study population. Of the 203 patients, 97 (47.8%) presented with stage I disease, 25 (12.3%) with stage II disease, 34 (16.7%) with stage III disease, and 37 (18.2%) with stage IV disease. Median follow-up time was 6.20 years (interquartile range 3.2–9.4). The 5-year overall survival (OS) rate after the surgery was 62.6%.

**Table 1:** Characteristics of the study population according to different Immunoscore groups.

Immune cell score	All	Low	Intermediate	High	*p*
Number of patients	*n* = 203 (%)	*n* = 38 (%)	*n* = 111 (%)	*n* = 54 (%)	
Age (mean)	66.5	67.7	65.9	67.0	0.423
Gender					0.094
Men	110 (54.2)	16 (42.1)	59 (53.2)	35 (64.8)	
Women	93 (45.8)	22 (57.9)	52 (46.8)	19 (35.2)	
Charlson Comorbidity Index					0.269
0	85 (41.9)	17 (44.7)	41 (36.9)	27 (50.0)	
1	58 (28.6)	11 (28.9)	38 (34.2)	9 (16.7)	
2	39 (19.2)	8 (21.1)	21 (18.9)	10 (18.5)	
≥ 3	21 (10.3)	2 (5.3)	11 (9.9)	8 (14.8)	
Surgery type					0.770
Open nephrectomy	140 (69.0)	28 (73.7)	73 (65.8)	39 (72.2)	
Open partial	22 (10.8)	4 (10.5)	15 (13.5)	3 (5.6)	
Laparoscopic nephrectomy	37 (18.2)	5 (13.2)	21 (18.9)	11 (20.4)	
Laparoscopic partial	4 (2.0)	1 (2.6)	2 (1.8)	1 (1.9)	
Pathological Stage					0.302
I	97 (47.8)	20 (52.6)	53 (47.7)	24 (44.4)	
II	25 (12.3)	3 (7.9)	14 (12.6)	8 (14.8)	
III	34 (16.7)	7 (18.4)	17 (15.3)	10 (18.5)	
IV	37 (18.2)	4 (10.5)	25 (22.5)	8 (14.8)	
Unknown	10 (4.9)	4 (10.5)	2 (1.8)	4 (7.4)	
T-stage					0.742
T1	112 (55.2)	23 (60.5)	60 (54.1)	29 (53.7)	
T2	39 (19.2)	4 (10.5)	23 (20.7)	12 (22.2)	
T3	44 (21.7)	9 (23.7)	25 (22.5)	10 (18.5)	
T4	4 (2.0)	0 (0.0)	3 (2.7)	1 (1.9)	
Unknown	4 (2.0)	2 (5.3)	0 (0.0)	2 (3.7)	
Fuhrman grade					0.349
I	38 (18.7)	7 (18.4)	24 (21.6)	7 (13.0)	
II	114 (56.2)	20 (52.6)	62 (55.9)	32 (59.3)	
III	39 (19.2)	7 (18.4)	22 (19.8)	10 (18.6)	
IV	12 (5.9)	4 (10.5)	3 (2.7)	5 (9.3)	

**Figure 1:**
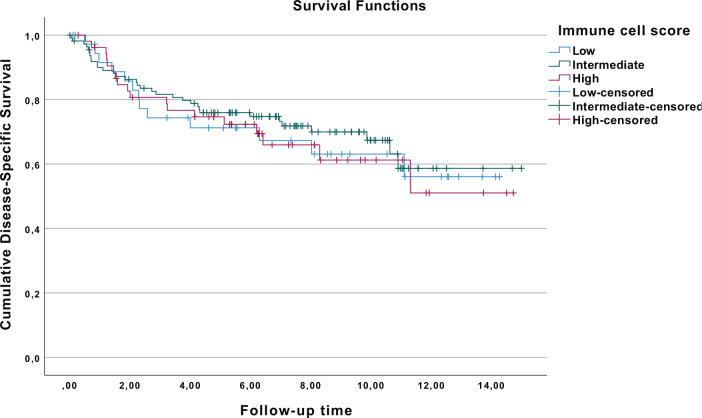
Kaplan–Meier curves for renal cell cancer patients with low-, intermediate-, and high-immune cell score.

For all patients, the 5-year disease-specific survival (DSS) rate after surgery was 74.7%. Based on the immune cell score, the patients were divided into three groups: low (*n* = 38), intermediate (*n* = 111), and high (*n* = 54). The 5-year DSS rates after surgery were 71.2%, 76.0%, and 74.6% for low-, intermediate-, and high-immune cell score groups, respectively. There were no differences in DSS among low-, intermediate-, and high-immune cell score groups (Figure 1).

In Cox regression analysis, after adjustment for confounders, the HRs for disease-specific mortality were 0.93 (95% CI 0.48–1.79) and 1.12 (0.52–2.37) for intermediate- and high-immune cell score groups when compared to low-immune cell score group (reference) (Table 2), respectively. CD3+ and CD8+ cell densities were also analyzed separately. In the analyses of CD3+ cell densities, there were no associations between CD3+ cell densities in the center of the tumor or in the invasive margin and disease-specific mortality (Table 2). Furthermore, similar results were seen with CD8+ cell densities in the center of the tumor or in the invasive margin, as the HRs were 0.85 (0.52–1.39) and 0.89 (0.54–1.46), respectively. Corresponding results were seen in the Kaplan–Meier analysis (Supplementary Figures 2 and 3). Moreover, when additional optimal cutoffs were searched with ROC curves, the result was still negative (Supplementary Figure 4).

**Table 2:** Hazard ratios (HRs) with 95% confidence intervals (CIs) of the disease-specific mortality in clear-cell renal cell cancer (ccRCC) patients with low-, intermediate-, and high-immune cell score and HRs with 95% CI of the disease-specific mortality in ccRCC patients for CD3+ and CD8+ cell densities in the invasive margin (IM) and center of tumor (CT).

	Unadjusted		Adjusted^a^	
	HR	95% CI	HR	95% CI
**Immunoscore**				
Low (reference)				
Intermediate	0.87	0.46–1.65	0.93	0.48–1.79
High	1.03	0.50–2.11	1.12	0.52–2.37
**CD3+ IM**				
Low (reference)				
High	1.14	0.69–1.88	1.18	0.70–1.98
**CD3+ CT**				
Low (reference)				
High	0.99	0.61–1.63	1.17	0.69–1.98
**CD8+ IM**				
Low (reference)				
High	0.89	0.54–1.46	0.93	0.56–1.54
**CD8+ CT**				
Low (reference)				
High	0.51	0.52–1.39	0.94	0.56–1.56


a Adjusted for Charlson Comorbidity Index, gender, and age.

## Discussion

The main finding of the current study is that there is no association between CD3+ and CD8+ T cell-based immune cell score and DSS among ccRCC patients who have undergone surgery. Furthermore, the separate components of immune cell score, namely, CD3+ and CD8+ lymphocyte densities, did not show associations with DSS, either. This highlights the need for a critical evaluation of whether the immune cell profile can be used as a prognostic tool in ccRCC.

Our findings add to the limited number of previous studies on the topic. Selvi et al. used retrospective data of 129 patients to determine the possible prognostic effect of ‘immunoscore’ in patients with ccRCC [14]. They found that favorable (high) ‘immunoscore’ was associated with prolonged disease-free survival (DFS), progression-free survival (PFS), and OS. Furthermore, Selvi et al. reported that favorable (high) ‘immunoscore’ was associated with prolonged OS but not with DFS and PFS in a small population (*N* = 48) of patients with a diagnosis of non-ccRCC following nephrectomy or partial nephrectomy [16]. Guo et al. more widely evaluated the tumor microenvironment, as they incorporated CD3+ T cells in the tumor center, CD4+FOXP3+CD45RO+ regulatory T cells in the tumor center, and CD8+PD1+ T cells in the invasive margin to build a novel immune feature-based score called ‘neo-immunoscore’ to predict the OS of ccRCC patients after nephrectomy [17]. This study included 82 retrospectively collected ccRCC patients. According to their results, ‘immunoscore’ based on CD3+ and CD8+ predicts favorable survival in ccRCC patients. However, the area under the receiver operator curve (AUC) for the ‘neo-immunoscore’ was higher than for the ‘immunoscore’. On the contrary, another study reported that increased infiltration of tumor tissue by T cells in RCC patients is associated with shorter survival, but the higher proliferative activity of CD8(+) T cells in contact with tumor cells is linked to longer survival, highlighting the importance of considering the effectiveness of antitumor immunity in immunotherapy approaches [18]. Furthermore, this observation offers a potential explanation for the relatively modest outcomes observed in our study.

Some additional studies have evaluated the distinct facets of the immune contexture of renal cell cancer. Giraldo et al. found that high peritumoral density of CD8+ cells is not associated with better prognosis in ccRCC patients like in several other solid cancer types [19]. Instead, the association between peritumoral CD8+ cells and prognosis depended on the expression of the immune checkpoints (PD-1, LAG-3, PD-L1, and PD-L2) and the localization of dendritic cells in the tumor microenvironment. In our study, immune checkpoints and dendritic cells were not determined, and we could not evaluate or validate these hypotheses. Using an algorithm based on gene expression (CIBERSORT), Zhang et al. assessed tumor-infiltrating immune cells among 538 ccRCC patients [20]. In their analyses, there was no association between cytotoxic T cells and survival among ccRCC patients. However, in chromophobe RCC, cytotoxic T cells were associated with favorable outcome. This suggests that the significance of immune cell infiltrates may also differ by the histological subtypes of RCC, which is a relevant topic for further research.

One strength of the study is a relatively long follow-up time, which allows us to reliably assess the association between the immune cell score and mortality. Another strength is the use of Finnish nationwide compulsory databases, which provide us a comprehensive and accurate death register. Additionally, our study group has experience in the analysis of immune infiltrates in other tumor types [21, 22], which supports the quality in both immunohistochemical and statistical analyses. A major limitation of our study is the relatively small sample size, which limits the statistical power and the use of subgroup analyses. Nevertheless, our sample size is bigger than in any previous study assessing the prognostic effect of T cell-based immune cell score in ccRCC patients. The patients included in the study have received oncological treatments according to national guidelines of that time. A lack of exact information concerning antitumoral treatments which patients have received is also one of the study limitations. ccRCC is known for its immunogenic nature, and the tumor microenvironment plays a crucial role in modulating the immune response [23]. However, the immune landscape within RCC tumors can be highly heterogeneous, with variations in immune infiltrates, checkpoint molecule expression, and immunomodulatory factors [24]. Therefore, a single marker or score may not capture the complex interplay between the tumor and the immune system. It is important to note that the immune cell score, including CD3+ or CD8+ T cells, may possess predictive value when jointly analyzed with immune modulators such as PD-1, LAG-3, PD-L1, and PD-L2. Nonetheless, before these methods can be deemed suitable for clinical practice, further studies of high quality are warranted.

In conclusion, CD3+ and CD8+ T cell density based immune cell score cannot be used as a prognostic tool in ccRCC patients based on the results of the current study. Furthermore, neither CD3+ nor CD8+ alone was associated with survival. Our results are controversial compared to the limited number of previous studies on the topic, and the reason for this difference is unclear. Therefore, large-scale studies specifically investigating more detailed immune cell infiltrates in patients with high-risk localized or locally advanced RCC are needed.

## Supplementary Material

CD3+ and CD8+ T cell-based immune cell score as a prognostic factor in clear-cell renal cell carcinoma

## Data Availability

Research data are not shared.
