# Supplementary figures and images for: CD3+ and CD8+ T cell-based immune cell score as a prognostic factor in clear-cell renal cell carcinoma
Source: Acta Oncol. 2024 Mar 28;63:19690. doi: 10.2340/1651-226X.2024.19690 (PMC11332446; doi:10.2340/1651-226X.2024.19690)

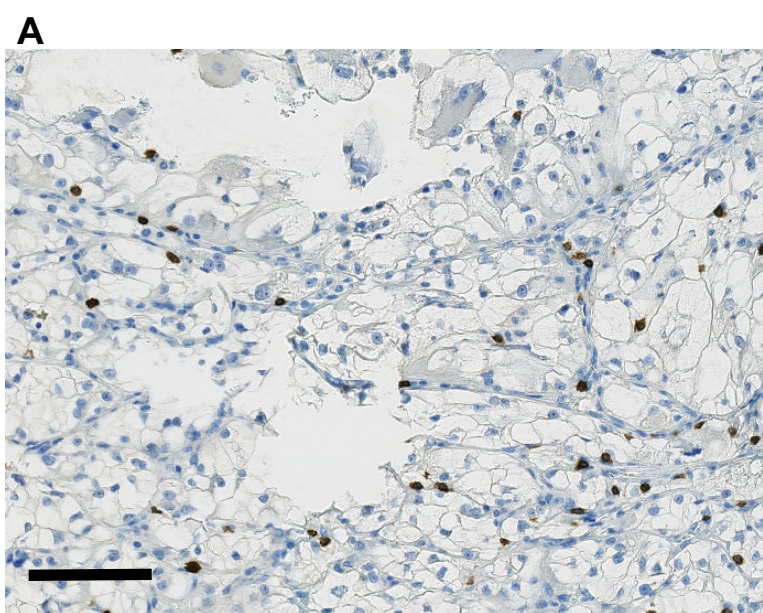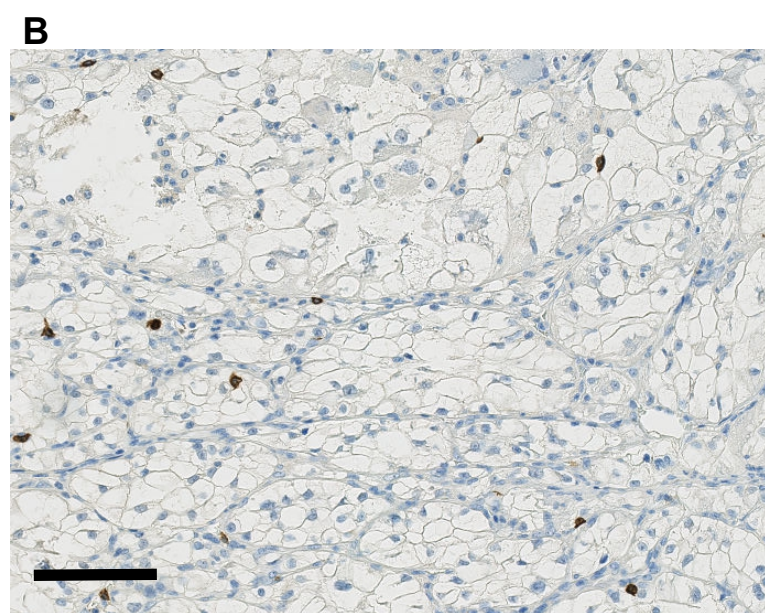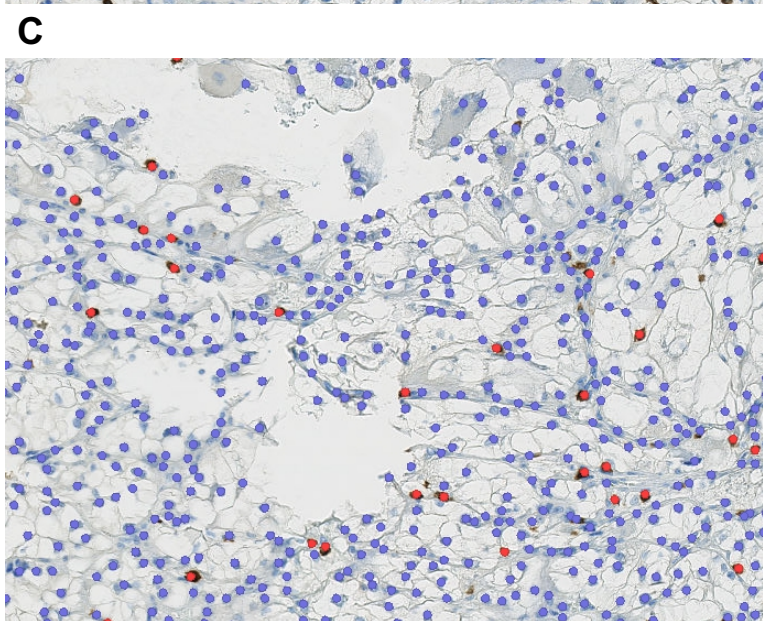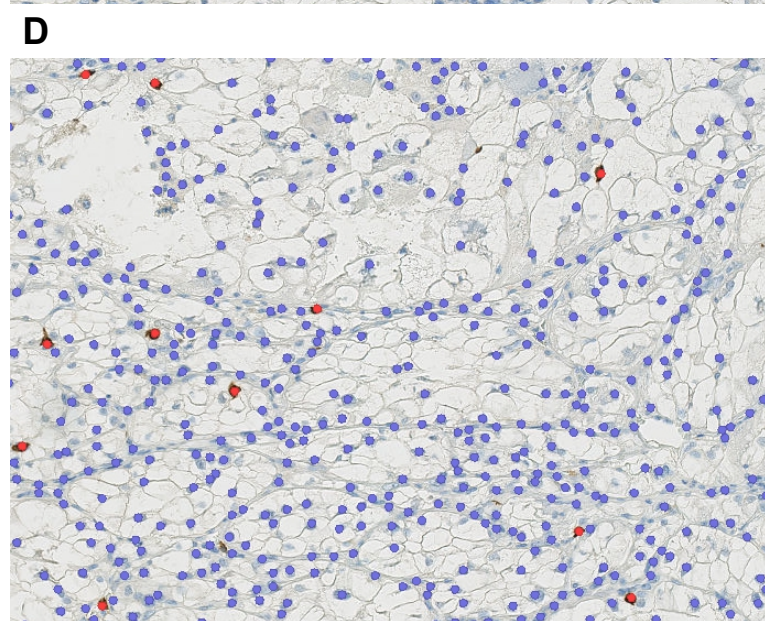

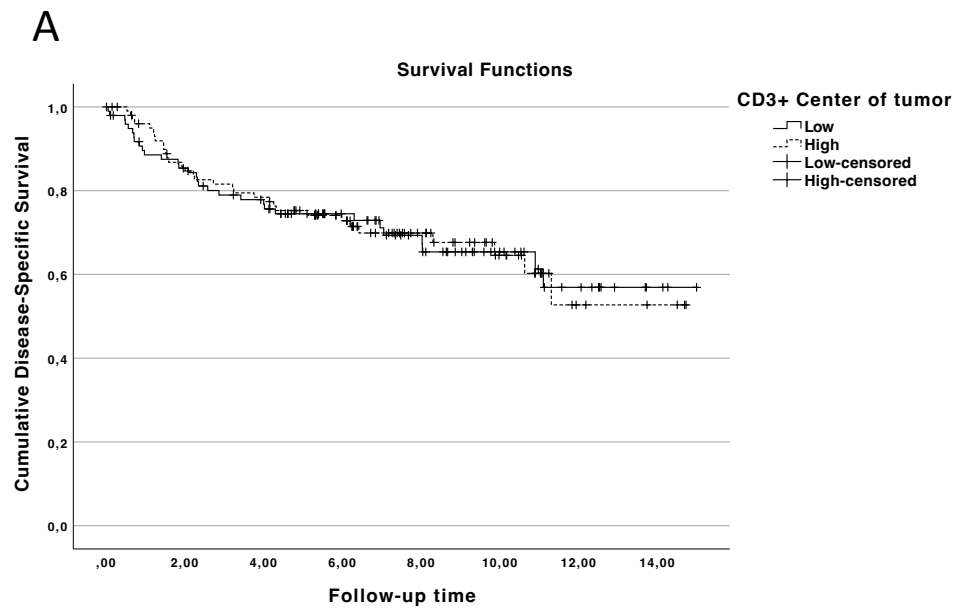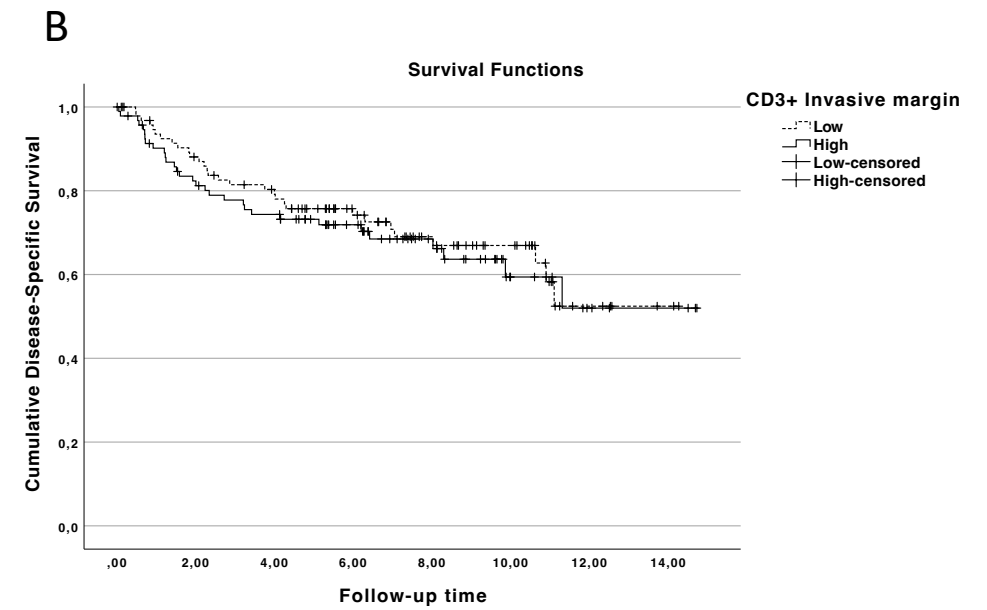

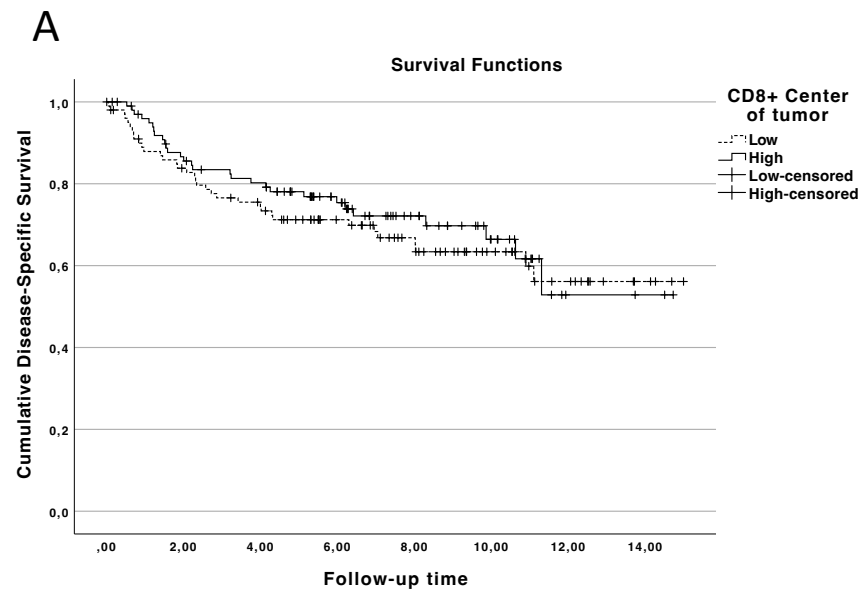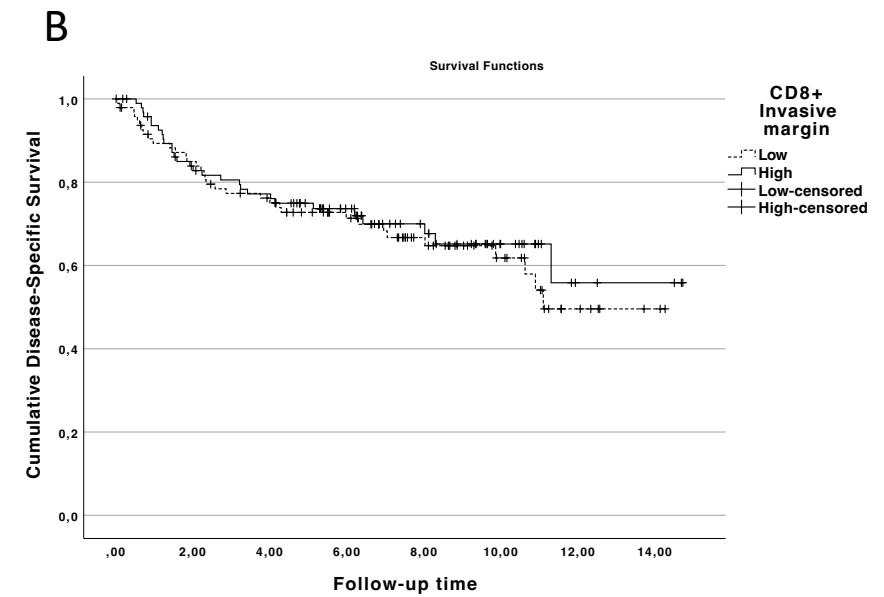

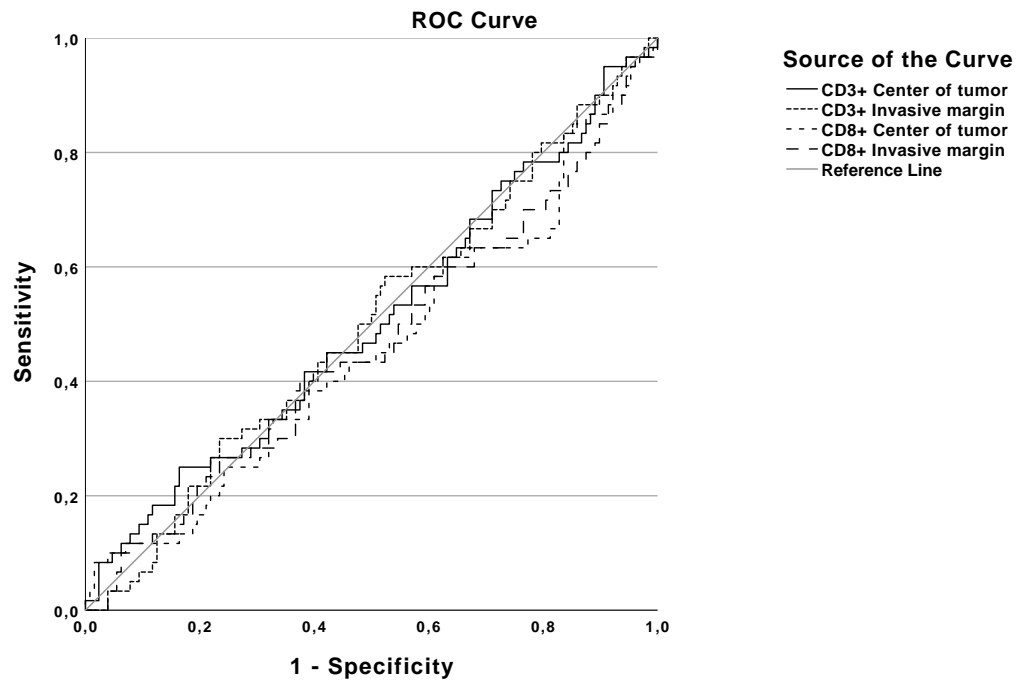

Supplement: CD3+ and CD8+ T cell-based immune cell score as a prognostic factor in clear-cell renal cell carcinoma [file AO-63-19690-s1.pdf]
